# VNTR analysis reveals unexpected genetic diversity within *Mycoplasma agalactiae*, the main causative agent of contagious agalactia

**DOI:** 10.1186/1471-2180-8-193

**Published:** 2008-11-07

**Authors:** Laura McAuliffe, Colin P Churchward, Joanna R Lawes, Guido Loria, Roger D Ayling, Robin AJ Nicholas

**Affiliations:** 1 Mycoplasma Group, Department of Statutory and Exotic Bacteria, Veterinary Laboratories Agency (Weybridge), Surrey, KT15 3NB, UK; 2 Istituto Zooprofilatico Sperimentale della Sicilia, 90129, Palermo, Sicily, Italy

## Abstract

**Background:**

*Mycoplasma agalactiae *is the main cause of contagious agalactia, a serious disease of sheep and goats, which has major clinical and economic impacts. Previous studies of *M. agalactiae *have shown it to be unusually homogeneous and there are currently no available epidemiological techniques which enable a high degree of strain differentiation.

**Results:**

We have developed variable number tandem repeat (VNTR) analysis using the sequenced genome of the *M. agalactiae *type strain PG2. The PG2 genome was found to be replete with tandem repeat sequences and 4 were chosen for further analysis. VNTR 5 was located within the hypothetical protein MAG6170 a predicted lipoprotein. VNTR 14 was intergenic between the hypothetical protein MAG3350 and the hypothetical protein MAG3340. VNTR 17 was intergenic between the hypothetical protein MAG4060 and the hypothetical protein MAG4070 and VNTR 19 spanned the 5' end of the pseudogene for a lipoprotein MAG4310 and the 3' end of the hypothetical lipoprotein MAG4320.

We have investigated the genetic diversity of 88 *M. agalactiae *isolates of wide geographic origin using VNTR analysis and compared it with pulsed field gel electrophoresis (PFGE) and random amplified polymorphic DNA (RAPD) analysis. Simpson's index of diversity was calculated to be 0.324 for PFGE and 0.574 for VNTR analysis. VNTR analysis revealed unexpected diversity within *M. agalactiae *with 9 different VNTR types discovered. Some correlation was found between geographical origin and the VNTR type of the isolates.

**Conclusion:**

VNTR analysis represents a useful, rapid first-line test for use in molecular epidemiological analysis of *M. agalactiae *for outbreak tracing and control.

## Background

*Mycoplasma agalactiae *is the main causative agent of contagious agalactia (CA), a syndrome that causes mastitis, arthritis, conjunctivitis and pneumonia in sheep and goats. *M. agalactiae *is prevalent worldwide but causes particular problems around the Mediterranean basin where it has a major clinical and economic impact on the small ruminant milk industry [1]. In most cases, infected hosts spontaneously recover from acute clinical signs within a few weeks but develop a chronic infection accompanied by shedding of *M. agalactiae *in milk and/or other body secretions for years without presenting any clinical signs [1]; these (asymptomatic) carriers can transmit the bacteria to other susceptible animals and cause acute disease [2]. This has seriously hindered the eradication of CA despite research into vaccines [3,4], identification of effective antibiotics [5,6] and improved diagnostic testing [7]. Strains of *M. agalactiae *exhibit very little genetic variation [8,9] while being antigenically diverse [8-11]. One of the reasons for this antigenic divergence is the presence of gene families of variable proteins such as Avg and VmpA; these are similar to the Vsp gene family in *Mycoplasma bovis *(*M. bovis*) [12-14].

As *M. agalactiae *shows differing prevalence across the world, and as it is currently absent from some countries, notably the UK, there is a pressing need for molecular epidemiological techniques which enable a high degree of strain differentiation allowing the tracing of the source of disease outbreaks.

Recently the genome of *M. agalactiae *has been sequenced making it highly amenable to analysis using newer typing methods such as variable number of tandem repeats (VNTR) analysis and multi locus sequence typing (MLST). VNTRs have been described for a variety of organisms including *Staphylococcus aureus *[15], *Mycobacterium tuberculosis *[16], *Borrelia *spp [17], *Leptospira *spp [18], *Brucella *spp [19], *Francisella tularensis *[20], *Legionella pneumophila *[21] and has recently been successfully applied to *M. mycoides *subspecies *mycoides *SC (*Mmm *SC) [22].

VNTRs are simple repeated DNA sequences that vary in copy number and have been shown to give a high level of discriminatory power providing information regarding both evolutionary and functional bacterial diversity [23]. With the increasing number of sequenced genomes and the development of VNTR databases, VNTR is an increasingly amenable technique for bacterial typing.

We describe the identification and characterisation of tandem repeats within the *M. agalactiae *PG2 genome, the selection of 4 VNTRs which showed most intraspecific variation, and their use as a molecular epidemiological tool for the analysis of 88 *M. agalactiae *strains. VNTR analysis was compared with PFGE and RAPD analysis. This study has analysed the largest numbers of strains of differing geographical locations of any previous studies and also used molecular epidemiological methods not previously used on *M. agalactiae *to determine the extent of their genetic diversity.

## Methods

### Culture of mycoplasmas

*M. agalactiae *isolates (as listed in additional file 1) were stored at -80°C and grown in 3 ml aliquots of Eaton's broth media [24] for 24 hours. A loop full of the culture was then plated on Eaton's solid media and incubated at 37°C, 5% CO_2 _for 48 or 72 hours. Single colonies were selected and transferred to 3 ml Eaton's broths and incubated until signs of growth, aliquots were then frozen in 15% (v/v) glycerol at -80°C until required.

### Confirmatory tests

DNA from each strain was extracted from 1.5 ml of culture using a Genelute gDNA extraction kit (Sigma) following the manufacturers instruction. To confirm the identity of the isolates *M. agalactiae *PCR based on the *uvr*C gene [25] and 16S rDNA PCR DGGE [7] were performed.

### Random amplified polymorphic DNA (RAPD)

RAPD was performed essentially as described previously [26]. Briefly, PCR reaction mixes were made up in 50 μl volumes containing: 15 mM Tris-HCl pH 8.0, 50 mM KCl, 2 mM MgCl_2_, 400 μM dNTPs, 0.8 μM of HUM-4 primer 5'-3' ACG GTA CAC T [27], 2.5 U of Taqgold (Applied Biosystems) and 1 μl of the test sample. The following thermocycling conditions used were; 5 minutes initial denaturing at 95°C followed by 40 cycles of 15 seconds of denaturing at 95°C, 1 minute of primer annealing at 37°C, and 1:30 minutes of primer extension; cycles were followed by a final extension time at 72°C for 5 minutes. Amplicons were separated by electrophoresis on 2% agarose gels against a 50–2000 bp amplisize DNA marker (Bio-Rad) followed by ethidium bromide staining. Gel images were analysed using Bionumerics (Applied Maths).

### Pulsed Field Gel Electrophoresis (PFGE)

Cells were grown in 20 ml Eaton's broth until late log phase and centrifuged at 4500 × g for 20 minutes at 4°C and washed three times in 0.1 M PBS + 10% glucose (PBSG). Cells were then resuspended in 1 ml of PBSG and 60 μl of the cell suspension added to an equal volume of 2% agarose and allowed to set in a plug mould. Once set, the plugs were added to lysis buffer (10 mM Tris-HCl, 1 mM EDTA + 1% N-lauryl sarcosine + 1 mg/ml proteinase K) and incubated at 45°C for 48 hours. Lysis buffer was then removed by five washes of TE (10 mM Tris-HCl + 1 mM EDTA) and plugs finally stored in TE at 4°C until required. A three millimetre slice of each plug was digested with 20 U of *Sma*I (Promega) overnight at room temperature following manufacturers instruction. Digested slices and a 0.1–200 Kb pulse marker (Sigma) were loaded on to 1% pulsed field agarose gels and ran in a BIO-RAD CHEF III system in 0.5 × TBE for 18 hours using pulse times of 4 to 40 seconds and a constant buffer temperature 14°C. After electrophoresis, gels were stained in ethidium bromide for 20 minutes and then destained for up to 16 hours in distilled water. Gel pictures were taken using a transilluminator and pictures analysed using BioNumerics (Applied Maths).

### Tandem repeat identification and VNTR analysis

Tandem repeats in the published *M. agalactiae *genome were identified using the Tandem repeat finder [28] hosted at . Primers were designed for the region flanking each VNTR using Primer3  and primers were tested using BLAST (NCBI) within the *M. agalactiae *genome to confirm that they would only bind in the region flanking the VNTR of interest.

VNTR sequences were amplified using the primer pairs listed in table 1. For the PCR, 1 μl of DNA was added as a template to 49 μl of a reaction mixture containing 10 mM Tris-HCl (pH 9.0), 1.5 mM MgCl_2_, 50 mM KCl, 0.1% Triton X-100, 0.2 mM each deoxynucleoside triphosphate, and 0.5 U of Taqgold (Applied Biosystems). The cycling conditions were: denaturation at 94°C for 5 min followed by 30 cycles of 95°C for 1 min, 56°C for 45 sec and 72°C for 1 min, a final extension step of 72°C for 10 min and samples were kept at 4°C until analysis. Aliquots were checked for correct amplification by electrophoresis on 2.5% agarose gels followed by visualization with ethidium bromide under UV illumination.

**Table 1:** VNTRs used in this study, their genomic origin, description and primers used for amplification

Genome position (bp)	VNTR name	Unit length	Copy number	Percent matches	Total length	Primer sequence
733172–733400	Magal VNTR 5	24	2.3	72%	226	F-gaaagagaaaggaagctgaa R-ggatcattatcgctttttga
397220–397378	Magal VNTR 14	13	2.0	100%	158	F-ttgaaatatccgcttaagaaa R-aatttgcatttaatggtgct
477915–478124	Magal VNTR 17	14	3.6	72%	209	F-tttagcttttgattcaatactttc R-aaagaattatgcgagcattt
504535–504760	Magal VNTR 19	22	2.3	83%	153	F-ttgcttcttgtgcttctttt R-aaggggatcaaccagataat

VNTR profiles were recorded as character data using allelic profiles and then dendrograms were constructed with PHYLIP (the PHYLogeny Inference Package) hosted at  using neighbor-joining. Tree display and output was performed using Phylodendron.

### Calculation of Simpson's index of diversity

Simpson's index of diversity, which is based on the probability that two unrelated strains will be placed into different typing groups, was calculated according to Hunter and Gaston [29]. Simpson's index of diversity ranges from 0.0 to 1.0, where 0.0 means that all strains in a population are of an identical type and conversely, 1.0 indicates that all of the strains in a population are different can be distinguished from one other.

## Results

### Characterisation and position of tandem repeats within the *M. agalactiae *genome

In total 30 tandem repeats were detected within the *M. agalactiae *genome using the parameters of a maximum period size of 1000 bp, a minimum alignment of 20 bp and a minimum score of 50. However, many of these tandem repeats were present in only two copies and showed low internal conservation (less than 80%). Initial screens using 10 strains of wide geographical origin revealed that 7 tandem repeats showed differences between strains. 4 VNTRs were selected for further analysis and were tested against 88 strains. The properties of the VNTRs selected are described below and in table 1.

#### VNTR 5

Consensus sequence in *M. agalactiae *PG2:

GAGAAAAAGAAACAGGAAGAAGAA

Entire sequence in *M. agalactiae *PG2

GAGAAAAAGAAACAGGAAGAAGAAGAGAGAAAGAGAAAGGAAGCT

GAAGCAGAGAAA

Within the hypothetical protein MAG6170 a predicted lipoprotein.

#### VNTR 14

Consensus sequence in *M. agalactiae *PG2: TTTAGATTGCTAA

Entire sequence in *M. agalactiae *PG2: TTTAGATTGCTAATTTAGATTGCTAA

VNTR 14 is intergenic between the hypothetical protein MAG3350 and the hypothetical protein MAG3340.

#### VNTR 17

Consensus sequence in *M. agalactiae *PG2: TTCTATTTATTACC

Entire sequence in *M. agalactiae *PG2: TTCTATTTATTACCTTTTATATACCTTCTAGTTATTACCTTTATTA

VNTR 17 is intergenic between the hypothetical protein MAG4060 and the hypothetical protein MAG4070.

#### VNTR 19

Consensus sequence in *M. agalactiae*: CTTCTTCTTGCTTTCTTTTTTT

Entire sequence in *M. agalactiae *PG2: CTTCTTCTTGCTTTCTTTTTTTCTTCTTGTTGTTTTCTTTCTTCTTCTTTTG

VNTR 19 spans the 5' end of the pseudogene for a lipoprotein MAG4310 and the 3' end of the hypothetical lipoprotein MAG4320.

VNTR 5 was found to show some size variation between *M. agalactiae *isolates with strain L2 (designated group 2) having a slightly larger PCR product than other isolates (group 1).

VNTR 14 did not generate size variation between the isolates but was absent in some isolates, giving 2 groups, those with VNTR 14 (group 1) and those which lacked it (group 0). Group 1 contained isolates from a wide geographical origin with isolates from Spain, Greece, Macedonia, Sicily, Sardinia and Portugal all in this group. Group 0 isolates originated in Gran Canaria, Sicily, Sardinia and Greece.

**Figure 1:**
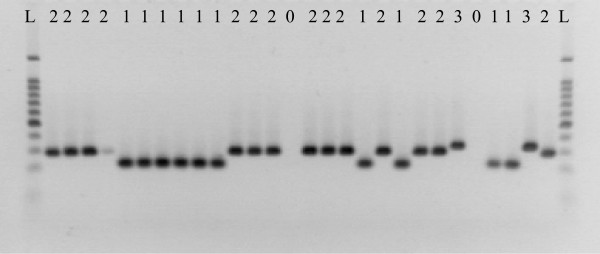
**Variation in fragment size as seen with VNTR 17.** L; Biorad 50–2000 bp, where 0, profile 0; 1, profile 1; 2, profile 2; 3, profile 3.

VNTR 17 generated 4 different profiles, group 0 lacked this VNTR and contained strain L9 from Gran Canaria, 281F03 from Spain and 227F06 from Greece. Group 1 contained 19 strains from a wide geographical origin with strains from the Spain, Italy, Greece, Sardinia. Group 2 contained 63 isolates from Sicily, Sardinia, mainland Italy, Macedonia, Portugal, Greece and Spain. Group 3 contained only 3 isolates, all of which were obtained from Greece. A representative gel image of VNTR 17 profiles is shown in figure 1.

VNTR 19 gave two different sized products when the 88 *M. agalactiae *strains were tested. Group 1 contained 82 strains with wide geographical origin from Macedonia, Portugal, Spain, Sardinia, Italy and Greece. Group 2 contained 7 isolates; 6 from Italy and a single strain from Sardinia.

**Figure 2:**
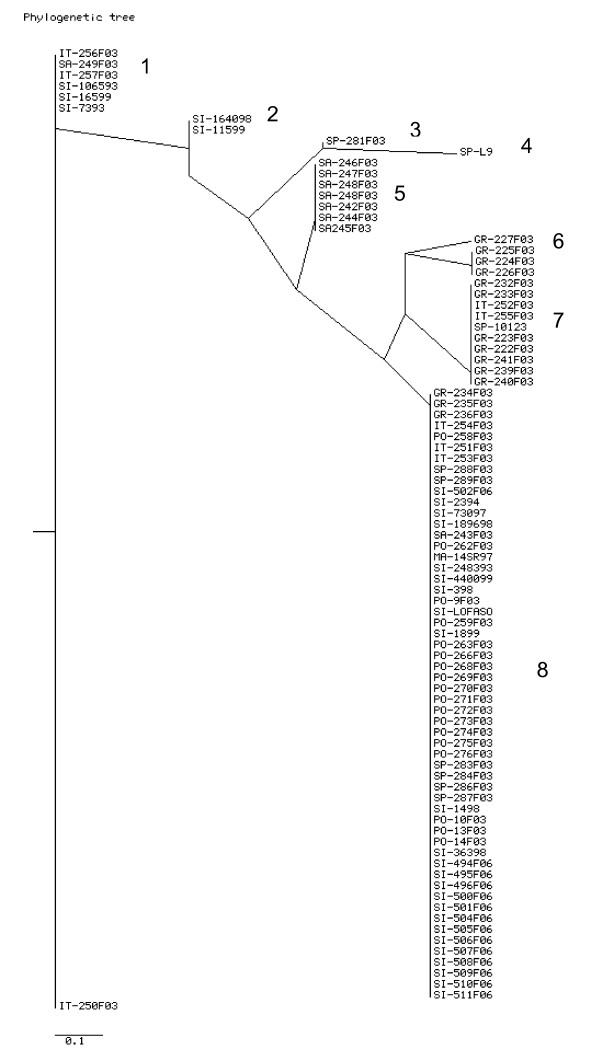
**Genetic relationships between *M. agalactiae *strains based on comparison of VNTR profile types**. The country of origin of the isolates is denoted by IT for mainland Italy, SI for Sicily, SA for Sardinia, GR for Greece, PO for Portugal and MA for Macedonia. The dendrogram was produced using the Neighbor-joining method of the Phylip program.

In total there were 9 different profiles obtained using VNTR analysis and these are summarised in additional file 1. The 9 different profiles could be divided into 8 different groups as shown in figure 2. There was some correlation between geographical origin and the 10 groups. Group 1 contained mainland Italian, Sicilian and a Sardinian strain, group 2 comprised of 2 Sicilian strains, group 3 contained a single Spanish strain, group 4 contained a single Spanish strain, groups 5 contained Sardinian strains, group 6 contained Greeks strains, group 7 contained predominantly Greek strains but 2 Italian strains were also found in this group and the type strain which originated in Spain. There were also a large group, group 8, which contained isolates of very diverse geographical origin with isolates present from Portugal, Spain, Italy, and Macedonia.

### PFGE analysis

**Figure 3:**
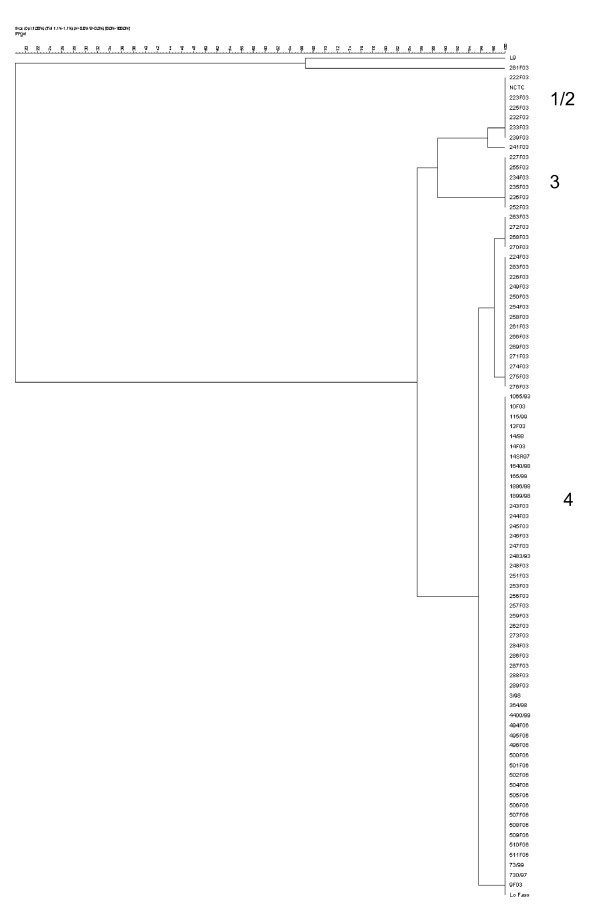
**Genetic relationships between *M. agalactiae *strains based on comparison of PFGE electrophoretic patterns. **The dendrogram was produced using the UPGMA method with DICE similarity coefficient.

**Figure 4:**
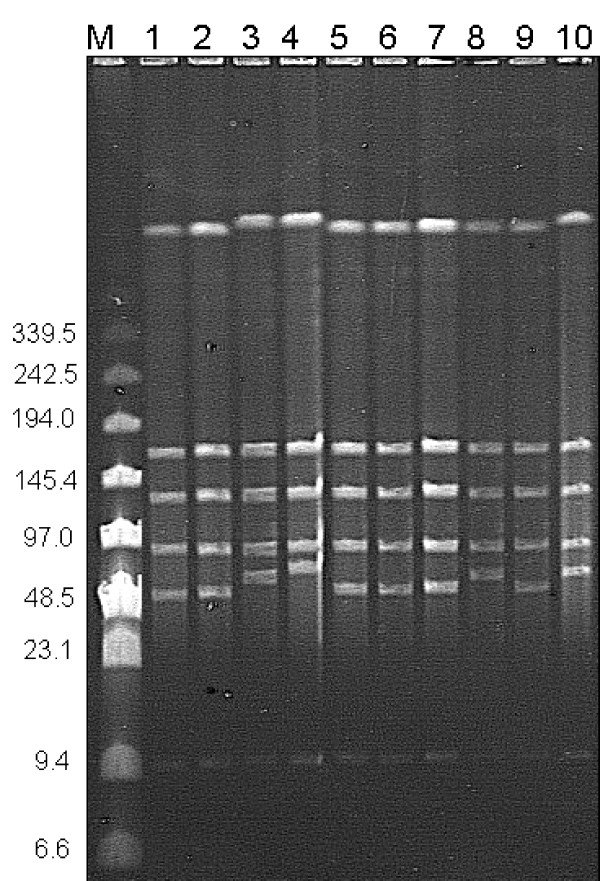
**Representative 1% pulsed-field gel of *Sma*I digested *M. agalactiae *DNA**. Lane M, BioRad pulse marker with fragment sizes in Kbp to the left; Lane 1, 730/97; lane 2, 1896/98; lane 3, 223F03; lane 4, 241F03; lane 5, 242F03; lane 6, 243F03; lane 7, Lo Faso; lane 8, NCTC 10123; Lane 9, 262F03 and lane 10, 237F03.

The restriction profiles of most of the strains were shown to be closely related with only some small differences in banding pattern. These profiles showed such close similarity that isolates were divided into 4 separate groups, group 1, 2, 3 and 4 based on a cut-off value of 80% (figure 3). The vast majority of isolates (69 of 86, 77.52%) fell into group 2 and gave largely indistinguishable profiles. There was some degree of congruence between the groups given using PFGE and the geographical origins of the isolates, Group 1 contained the single strain L9 from Gran Canaria which was unique as it produced four bands, group 2 contained another single Spanish strain 281F03 which unusually produced 5 bands, group 3 contained mainly Greek isolates and group 4 contained isolates of wide geographical origin including Portugal, Macedonia, Sardinia and Italy. Spanish isolates were particularly heterogeneous and were found in all 4 groups. Three strains were not typable using PFGE analysis. 84 of the 86 (98%) of strains tested produced six restriction fragments using *Sma*I. The fragments sizes were estimated to be 466, 174, 132, 91, 11 Kbp and a fragment that varied in size between strains of between 58–72 Kbp. A representative pulsed field gel is shown in figure 4.

### RAPD analysis

All strains tested produced 8–12 amplicons ranging in size of 0.5 and 1.5 Kb. Strain 81F03 gave completely different profiles from that of other *M. agalactiae *strains (71% and 69% similarity respectively giving a DICE stringency of 1.3% and a 1% level of optimisation) (results not shown). All other strains (87 out of 88, 98%) were within an 80% margin of similarity. Due to the variable and random nature of the technique, to validate reproducibility each strain was tested three times and because of the very small differences in banding pattern between isolates they clustered slightly differently on each repeat. Only isolates 281F03 and L9 were clearly different from all other isolates on each repeat. Due to the high level of genetic homogeneity and inconsistent grouping of strains by this typing method it would be impossible to subdivide these stains with any confidence.

### Congruence between typing techniques

There was a high degree of congruence between PFGE and VNTR analysis, with the exception of some strains in VNTR group 1121 which were split into either PFGE group 3 or group 4. VNTR analysis was capable of detecting much greater variation than PFGE analysis as many isolates found to be identical using PFGE were capable of being differentiated using VNTR analysis.

Simpson's index of diversity was calculated to be 0.324 for PFGE and 0.574 for VNTR analysis.

## Discussion

This study represents the first to apply VNTR analysis to *M. agalactiae*. Previous studies have shown that *M. agalactiae *is largely genetically homogeneous but may be subject to some degree of antigenic variation [8,9]. Analysis of 81 Italian isolates found that they were all completely homogeneous with no variation seen using PFGE analysis [8]. Insertion sequence analysis of *M. agalactiae *isolates with wide geographical origin found some degree of variation with some isolates containing the IS element IS*Mag *and other isolates lacking the element however it was not possible to subtype isolates using this method [30].

In our study, RAPD analysis was found to be unreliable for typing *M. agalactiae *but PFGE was capable of dividing these largely homogeneous isolates into 4 groups. VNTR analysis was however far more effective and enabled us to detect variation between *M. agalactiae *strains with the 88 isolates being divided into 9 VNTR types showing some correlation between geographical origin and the VNTR profiles of the isolates. Sardinian and Sicilian isolates were particularly interesting as they were the most diverse with isolates in many different VNTR groups whereas Portuguese isolates were found only within one group of wide geographical origin (and did not have their own distinct group). Isolates from mainland Italy were also found in many groups and did not fall into a group of distinct geographical origin. These groupings may reflect the degree of international trade of these countries with countries that are largely self sufficient with little import showing less heterogeneity in *M. agalactiae *isolates. Although we believe that the groupings generated using VNTR analysis are significant and reliable, some care must be taken when drawing inferences from a typing scheme based on a small number of VNTRs and that it would be have been interesting to include further VNTRs in the study had variation been detected in any other VNTRs.

Although mycoplasmas possess a reduced genome with only the genes necessary for replication and survival, they also possess a relatively large proportion of repetitive DNA, For example, it is known that for some other *Mycoplasma *species, such as *Mmm *SC, the genome contains as much as 29% repetitive DNA [31]. Therefore, it seems feasible that typing schemes based on analysis of repetitive DNA may be useful for *Mycoplasma *species.

*M. agalactiae *is closely related to the cattle pathogen *M. bovis *which was at one time a subspecies of *M. agalactiae *[32]. The *M. agalactiae *population structure, however appears to be dramatically different as it is subject to little intraspecific variation compared with *M. bovis *which was found to be highly heterogeneous when analysed using AFLP, RAPD and PFGE [26]. This difference in genetic diversity may reflect the distribution and movements of their respective hosts. Whereas *M. bovis *is found in cattle which are generally traded frequently over wide areas, *M. agalactiae *is found in sheep which are not subjected to the same degree of stock movement. Therefore, there is greater opportunity for genetic exchange between *M. bovis *isolates leading to a more heterogeneous population structure compared with *M. agalactiae*. The two *Mycoplasma *species may also differ in genetic diversity because of fundamental genetic differences, for example, *M. bovis *contains a greater number of insertion sequences than *M. agalactiae*.

Recent evidence has shown that *M. agalactiae *may have undergone horizontal gene transfer (HGT) [33,34]. As mycoplasmas have a reduced genome HGT is likely to be important as it enables mollicutes to rapidly adapt by a 'quantum leap' in evolution and enables genes for a certain advantageous phenotype to be transferred in a single block rather than by random point mutation and subsequent selection of strains with advantageous traits [35]. Interestingly some of the VNTRs found in this study are within regions of the genome which are prone to variation and involved in HGT. The pseudogene, MAG4310, in which VNTR 19 was found, has been identified as being a predicted lipoprotein which has undergone horizontal gene transfer with members of the *M. mycoides *cluster. This pseudogene may constitute a reservoir of sequence whose purpose is to generate surface variability [34] and hence it is not surprising that this region shows variation between *M. agalactiae *strains. Another VNTR, VNTR 17, which was found in the majority of strains tested, is in the region of the pseudogene MAG4060 which has been shown to be a pseudogene of an integrative conjugative element (ICE) which is involved in HGT.

The ICE in *M. agalactiae *is a novel mobile genetic element [33], homologous to that seen in *M. fermentans *[36], which spreads from donor to recipient cells by conjugation and exists in both chromosomal and free circular forms [33]. In this study we have demonstrated that conjugative elements, such as those seen within VNTR17, are widespread in representative strains of *M. agalactiae *but that the ICEs that encode these conjugative systems show significant sequence variation between strains.

## Conclusion

VNTR analysis provides a rapid, simple, molecular typing technique that can enable the differentiation of *M. agalactiae *strains and may provide a method of outbreak tracing. VNTR is also useful as it does not require the culture of isolates and could, in theory, be used on infections involving mixed *M. agalactiae *strains. Finally it has the advantage when compared with many traditional typing techniques that it is highly amenable to interlaboratory comparison and does not require expensive or complex automated analysis.

## Authors' contributions

LM designed the study, designed the VNTR system, drafted the manuscript and analysed results. CPC carried out PFGE and RAPD analysis. JRL carried out some of the VNTR analysis. GL provided strains and was part of initial PFGE studies. RDA obtained funding for this study. RAJN helped interpret results and critically revised the manuscript.

## Supplementary Material

Additional file 1 **Summary table of strains used in this study, VNTR profile and PFGE grouping.** Click here for file
