# Supplementary material for: VNTR analysis reveals unexpected genetic diversity within Mycoplasma agalactiae, the main causative agent of contagious agalactia
Source: BMC Microbiol. 2008 Nov 7;8:193. doi: 10.1186/1471-2180-8-193 (PMC2585094; doi:10.1186/1471-2180-8-193)
Supplement: Additional file 1 — Summary table of strains used in this study, VNTR profile and PFGE grouping. [file 1471-2180-8-193-S1.doc]

Summary table of strains used in this study, VNTR profile and PFGE grouping.

| Strain | Origin | Year | VNTR 5 | VNTR 14 | VNTR 17 | VNTR 19 | PFGE |
| --- | --- | --- | --- | --- | --- | --- | --- |
| 281F03 | Spain | 2003 | 1 | 0 | 0 | 1 | 2 |
| 250F03 | Italy | 2003 | 1 | 0 | 1 | 2 | 4 |
| 256F03 | Italy | 2003 | 1 | 0 | 1 | 2 | 4 |
| 249F03 | Sardina | 2003 | 1 | 0 | 1 | 2 | 4 |
| 257F03 | Italy | 2003 | 1 | 0 | 1 | 2 | 4 |
| 1065/93 | Sicily, Italy | 1993 | 1 | 0 | 1 | 2 | 4 |
| 1640/98 | Sicily, Italy | 1998 | 1 | 0 | 1 | 1 | 4 |
| 165/99 | Sicily, Italy | 1999 | 1 | 0 | 1 | 2 | 4 |
| 73/93 | Sicily, Italy | 1993 | 1 | 0 | 1 | 2 | 4 |
| 115/99 | Sicily, Italy | 1999 | 1 | 0 | 1 | 1 | 4 |
| 246F03 | Sardina | 2003 | 1 | 0 | 2 | 1 | 4 |
| 247F03 | Sardina | 2003 | 1 | 0 | 2 | 1 | 4 |
| 248F03 | Sardina | 2003 | 1 | 0 | 2 | 1 | 4 |
| 248F03 | Sardina | 2003 | 1 | 0 | 2 | 1 | 4 |
| 242F03 | Sardina | 2003 | 1 | 0 | 2 | 1 | ND |
| 244F03 | Sardina | 2003 | 1 | 0 | 2 | 1 | 4 |
| 245F03 | Sardina | 2003 | 1 | 0 | 2 | 1 | 4 |
| 227F03 | Greece | 2003 | 1 | 1 | 0 | 1 | 3 |
| 232F03 | Greece | 2003 | 1 | 1 | 1 | 1 | 3 |
| 233F03 | Greece | 2003 | 1 | 1 | 1 | 1 | 3 |
| 252F03 | Italy | 2003 | 1 | 1 | 1 | 1 | 3 |
| 255F03 | Italy | 2003 | 1 | 1 | 1 | 1 | 3 |
| NCTC 10123 | Spain | 1952 | 1 | 1 | 1 | 1 | 3 |
| 223F03 | Greece | 2003 | 1 | 1 | 1 | 1 | 3 |
| 222F03 | Greece | 2003 | 1 | 1 | 1 | 1 | 3 |
| 241F03 | Greece | 2003 | 1 | 1 | 1 | 1 | 3 |
| 239F03 | Greece | 2003 | 1 | 1 | 1 | 1 | 3 |
| 240F03 | Greece | 2003 | 1 | 1 | 1 | 1 | ND |
| 234F03 | Greece | 2003 | 1 | 1 | 2 | 1 | 3 |
| 235F03 | Greece | 2003 | 1 | 1 | 2 | 1 | 3 |
| 236F03 | Greece | 2003 | 1 | 1 | 2 | 1 | 3 |
| 254F03 | Italy | 2003 | 1 | 1 | 2 | 1 | 4 |
| 258F03 | Portugal | 2003 | 1 | 1 | 2 | 1 | 4 |
| 251F03 | Italy | 2003 | 1 | 1 | 2 | 1 | 4 |
| 253F03 | Italy | 2003 | 1 | 1 | 2 | 1 | 4 |
| 288F03 | Spain | 2003 | 1 | 1 | 2 | 1 | 4 |
| 289F03 | Spain | 2003 | 1 | 1 | 2 | 1 | 4 |
| 502F06 | Sicily, Italy | 2006 | 1 | 1 | 2 | 1 | 4 |
| 23/94 | Sicily, Italy | 1994 | 1 | 1 | 2 | 1 | ND |
| 730/97 | Sicily, Italy | 1997 | 1 | 1 | 2 | 1 | 4 |
| 1896/98 | Sicily, Italy | 1998 | 1 | 1 | 2 | 1 | 4 |
| 243F03 | Sardina | 2003 | 1 | 1 | 2 | 1 | 4 |
| 262F03 | Portugal | 2003 | 1 | 1 | 2 | 1 | 4 |
| 14SR97 | Macedonia | 1997 | 1 | 1 | 2 | 1 | 4 |
| 2483/93 | Sicily, Italy | 1993 | 1 | 1 | 2 | 1 | 4 |
| 4400/99 | Sicily, Italy | 1999 | 1 | 1 | 2 | 1 | 4 |
| 3/98 | Sicily, Italy | 1998 | 1 | 1 | 2 | 1 | 4 |
| 9F03 | Portugal | 1999 | 1 | 1 | 2 | 1 | 4 |
| Lo Faso | Sicily, Italy | 2000 | 1 | 1 | 2 | 1 | 4 |
| 259F03 | Portugal | 2003 | 1 | 1 | 2 | 1 | 4 |
| 1899 | Sicily, Italy | 1998 | 1 | 1 | 2 | 1 | 4 |
| 263F03 | Portugal | 2003 | 1 | 1 | 2 | 1 | 4 |
| 266F03 | Portugal | 2003 | 1 | 1 | 2 | 1 | 4 |
| 268F03 | Portugal | 2003 | 1 | 1 | 2 | 1 | 4 |
| 269F03 | Portugal | 2003 | 1 | 1 | 2 | 1 | 4 |
| 270F03 | Portugal | 2003 | 1 | 1 | 2 | 1 | 4 |
| 271F03 | Portugal | 2003 | 1 | 1 | 2 | 1 | 4 |
| 272F03 | Portugal | 2003 | 1 | 1 | 2 | 1 | 4 |
| 273F03 | Portugal | 2003 | 1 | 1 | 2 | 1 | 4 |
| 274F03 | Portugal | 2003 | 1 | 1 | 2 | 1 | 4 |
| 275F03 | Portugal | 2003 | 1 | 1 | 2 | 1 | 4 |
| 276F03 | Portugal | 2003 | 1 | 1 | 2 | 1 | 4 |
| 283F03 | Spain | 2003 | 1 | 1 | 2 | 1 | ND |
| 284F03 | Spain | 2003 | 1 | 1 | 2 | 1 | 4 |
| 286F03 | Spain | 2003 | 1 | 1 | 2 | 1 | 4 |
| 287F03 | Spain | 2003 | 1 | 1 | 2 | 1 | 4 |
| 14/98 | Sicily, Italy | 1999 | 1 | 1 | 2 | 1 | 4 |
| 10F03 | Portugal | 2003 | 1 | 1 | 2 | 1 | 4 |
| 13F03 | Portugal | 2003 | 1 | 1 | 2 | 1 | 4 |
| 14F03 | Portugal | 2003 | 1 | 1 | 2 | 1 | 4 |
| 363/98 | Sicily, Italy | 1998 | 1 | 1 | 2 | 1 | 4 |
| 494F06 | Sicily, Italy | 2006 | 1 | 1 | 2 | 1 | 4 |
| 495F06 | Sicily, Italy | 2006 | 1 | 1 | 2 | 1 | 4 |
| 496F06 | Sicily, Italy | 2006 | 1 | 1 | 2 | 1 | 4 |
| 500F06 | Sicily, Italy | 2006 | 1 | 1 | 2 | 1 | 4 |
| 501F06 | Sicily, Italy | 2006 | 1 | 1 | 2 | 1 | 4 |
| 504F06 | Sicily, Italy | 2006 | 1 | 1 | 2 | 1 | 4 |
| 505F06 | Sicily, Italy | 2006 | 1 | 1 | 2 | 1 | 4 |
| 506F06 | Sicily, Italy | 2006 | 1 | 1 | 2 | 1 | 4 |
| 507F06 | Sicily, Italy | 2006 | 1 | 1 | 2 | 1 | 4 |
| 508F06 | Sicily, Italy | 2006 | 1 | 1 | 2 | 1 | 4 |
| 509F06 | Sicily, Italy | 2006 | 1 | 1 | 2 | 1 | 4 |
| 510F06 | Sicily, Italy | 2006 | 1 | 1 | 2 | 1 | 4 |
| 511F06 | Sicily, Italy | 2006 | 1 | 1 | 2 | 1 | 4 |
| 225F03 | Greece | 2003 | 1 | 1 | 3 | 1 | 3 |
| 224F03 | Greece | 2003 | 1 | 1 | 3 | 1 | 4 |
| 226F03 | Greece | 2003 | 1 | 1 | 3 | 1 | ND |
| L9 | Gran Canaria, Spain |  | 2 | 0 | 0 | 1 | 1 |
